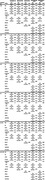# Evaluating the Role of Kidney Function and Comorbidities in Observed Racial/Ethnic Differences in Blood‐based Biomarkers of Neurodegeneration: Results from the Einstein Aging Study (EAS)

**DOI:** 10.1002/alz.092062

**Published:** 2025-01-09

**Authors:** Angel Garcia De La Garza, Cuiling Wang, Carol A. Derby, Richard B. Lipton, Henrik Zetterberg, Christopher G. Engeland, Mindy J. Katz

**Affiliations:** ^1^ Albert Einstein College of Medicine, Bronx, NY USA; ^2^ Department of Neurology, and Department of Epidemiology and Population Health, Albert Einstein College of Medicine, Bronx, NY USA; ^3^ UK Dementia Research Institute at UCL, London United Kingdom; ^4^ University of Gothenburg, Mölndal, Gothenburg Sweden; ^5^ The Pennsylvania State University, University Park, PA USA

## Abstract

**Background:**

Recent research reveals that Alzheimer’s Disease blood‐based biomarkers (BBB) are influenced by demographics as well as kidney function and comorbidities. Data on differences in BBBs by race and ethnicity are sparse. We examined whether racial/ethnic differences in BBBs persist after controlling for kidney function and comorbidities.

**Methods:**

Analyses included data from 274 EAS participants (mean age 77.57, SD 5.01, 67.5% Female, 46.4% Non‐Hispanic‐White, 41.2% Non‐Hispanic Black, 12.4% Hispanic). BBBs included β‐amyloid (Aβ40, Aβ42), Aβ42:Aβ40 ratio, pTau181, neurofilament light (NfL), and glial fibrillary acidic protein (GFAP). Estimated glomerular filtration rate (eGFR) was calculated using either age‐sex‐race‐adjusted (eGFR‐ASR) or age‐sex‐adjusted (eGFR‐AS) creatinine equations (Inker et al., 2021 NEJM). History of hypertension, diabetes, stroke, myocardial infarction, angina, and congestive heart failure were ascertained by self‐report. Linear regression was used to assesses race/ethnic differences in BBBs adjusted for age, sex, BMI, and MCI status. Further models adjusted for eGFR‐AS, eGFR‐ASR, and comorbidities. Non‐Hispanic Whites were treated as the reference group.

**Results:**

Initial models indicated significantly lower Aβ40 in non‐Hispanic Black and Hispanic (global p = 0.014) individuals and lower Aβ42 in non‐Hispanic Black (global p = 0.005) individuals compared to non‐Hispanic Whites. Further adjustment for using eGFR‐ASR attenuated racial/ethnic differences for Aβ40 (p = 0.099) and Aβ42 (p = 0.038), while adjustment using eGFR‐AS did not. We observed lower levels of NfL in non‐Hispanic Blacks and Hispanics (p = 0.003) adjusting for age, sex, BMI, and eGFR‐AS but not when adjusting for eGFR‐ASR. Both eGFR‐ASR and eGFR‐AS were negatively significantly associated with all BBBs. None of the comorbidities were significantly associated with any of the BBBs, and adjusting for comorbidities did not impact the observed racial/ethnic differences.

**Conclusion:**

Race/ethnic differences in Aβ40 and Aβ42 levels persisted after adjusting for eGFR‐AS and comorbidities. Race differences in BBBs were reduced in magnitude when adjusting for eGFR‐ASR, but not when adjusting for eGFR‐AS. Future research should pursue adjustment using the more accurate cystatin‐C eGFR equation to determine whether race differences persist. Understanding these biomarker level differences across various races/ethnicities is crucial for the future of personalized and effective diagnosis, progression monitoring, and treatment of Alzheimer's Disease.